# High-precision, non-invasive anti-microvascular approach via concurrent ultrasound and laser irradiation

**DOI:** 10.1038/srep40243

**Published:** 2017-01-11

**Authors:** Zizhong Hu, Haonan Zhang, Aghapi Mordovanakis, Yannis M. Paulus, Qinghuai Liu, Xueding Wang, Xinmai Yang

**Affiliations:** 1 Department of Biomedical Engineering, University of Michigan, Ann Arbor, MI, USA; 2 Department of Ophthalmology and Visual Sciences, University of Michigan, Ann Arbor, MI, USA; 3 Department of Ophthalmology, the First Affiliated Hospital of Nanjing Medical University, Nanjing, P.R. China; 4 Department of Radiology, University of Michigan, Ann Arbor, MI, USA; 5 Institute of Acoustics, Tongji University, Shanghai, P.R. China; 6 Bioengineering Research Center and Department of Mechanical Engineering, University of Kansas, Lawrence, KS, USA

## Abstract

Antivascular therapy represents a proven strategy to treat angiogenesis. By applying synchronized ultrasound bursts and nanosecond laser irradiation, we developed a novel, selective, non-invasive, localized antivascular method, termed photo-mediated ultrasound therapy (PUT). PUT takes advantage of the high native optical contrast among biological tissues and can treat microvessels without causing collateral damage to the surrounding tissue. In a chicken yolk sac membrane model, under the same ultrasound parameters (1 MHz at 0.45 MPa and 10 Hz with 10% duty cycle), PUT with 4 mJ/cm^2^ and 6 mJ/cm^2^ laser fluence induced 51% (p = 0.001) and 37% (p = 0.018) vessel diameter reductions respectively. With 8 mJ/cm^2^ laser fluence, PUT would yield vessel disruption (90%, p < 0.01). Selectivity of PUT was demonstrated by utilizing laser wavelengths at 578 nm or 650 nm, where PUT selectively shrank veins or occluded arteries. In a rabbit ear model, PUT induced a 68.5% reduction in blood perfusion after 7 days (p < 0.001) without damaging the surrounding cells. *In vitro* experiments in human blood suggested that cavitation may play a role in PUT. In conclusion, PUT holds significant promise as a novel non-invasive antivascular method with the capability to precisely target blood vessels.

Angiogenesis plays a critical role in the pathogenesis of numerous pathological conditions, including cancer, inflammation, and eye diseases. Significant research efforts have therefore been focused on the development of antivascular therapies, which include anti-angiogenic and vascular targeting therapies^1^. Whereas anti-angiogenic therapy aims to inhibit the growth of new vessels, vascular targeting therapy is designed to selectively destroy existing vessels. For example, angiogenesis inhibitors targeting the vascular endothelial growth factor (VEGF) signaling pathway have been developed to treat tumors and eye diseases^2^,^3^,^4^,^5^,^6^,^7^,^8^,^9^,^10^. While these treatments have proven to be efficacious, like other pharmaceutical approaches, the systemic administration of drugs can cause serious adverse effects including stroke and heart attack^11^,^12^, and patients may become drug-resistant^13^,^14^. Moreover, several studies on cancer have shown that while there is an initial antitumor effects, relapse and progressive tumor growth often follows after the use of angiogenesis inhibitors^2^,^15^,^16^,^17^. Local administration of these pharmaceuticals, e.g. intravitreal injection, also carries local risks, including local infection (endophthalmitis), cataract, and retinal detachment.

Additional antivascular approaches include photodynamic therapy (PDT)^18^,^19^,^20^,^21^, embolotherapy^22^,^23^,^24^,^25^, antivascular ultrasound therapy (AVUT)^26^,^27^,^28^, and photothermolysis^29^. These approaches have been developed to shut down or destroy existing blood vessels. Each of these methods has limitations affecting its utility, including being invasive in all three vascular-selective methods. PDT requires the systemic injection of photosensitizers, making the skin and eyes sensitive to light after the treatment. PDT may also cause burns, swelling, pain, and scarring in nearby healthy tissue and if it extravasates during intravenous injection. PDT in ophthalmic applications has also been associated with choroidal infarction and acute, severe vision loss^30^.

Embolotherapy, on the other hand, is an invasive procedure involving inserting a catheter through the vascular system to the origin of the vessel to be occluded, and then injecting an agent. This is performed to control bleeding, close fistulae or arteriovenous malformations, devascularize organs, and reduce tumors or varicoceles.

AVUT has demonstrated the capability of removing microvessels in murine tumors^26^,^27^,^28^ with the assistance of microbubbles, via disruption or embolization. However, similar to PDT and embolotherapy, AVUT requires the systemic injection of foreign particles (bubbles) into the blood stream, which leads to serious concerns of toxicity, efficiency, and emboli formation. In addition, very strict treatment time window is also a major concern in clinical adaptions of both PDT and AVUT because the circulation time of photosensitizers and microbubbles is limited. This limits the clinical applicability, particularly in high volume retinal practices, where PDT caused a significant disruption in clinic flow.

Photothermolysis is a selective therapeutic technique based on optical absorption to remove tissue. It does not necessarily target vasculature. By using milli- or micro-second laser pulses, photothermolysis can remove highly optically absorptive targets such as pigmented structures and cells. However, photothermolysis requires high laser energy. Because it relies on the thermal effect, clinical application of the excimer photothermolysis laser is limited to very superficial surface layer.

We developed, for the first time, a novel, noninvasive antivascular therapy that we call photo-mediated ultrasound therapy (PUT). PUT applies synchronized laser pulses and ultrasound bursts on target tissue, offering high-precision localized treatment of microvessels. The technique is based on controlled induction and promotion of micro-cavitation activity in the target vessels. When a laser pulse is absorbed by hemoglobin, the tissue is excited and the photoacoustic effect occurs. The concurrent application of ultrasound significantly increases the likelihood of micro-cavitation in the blood vessel^31^. The produced cavitation can then be further enhanced and driven by the subsequent ultrasound pulses. The shear stresses and microjets produced by oscillating bubbles in a microvessel can directly impact the physiological functions of endothelial cells, red blood cells, and platelets. This results in vasocontraction, blood clot formation, and hemorrhage^26^,^27^,^32^,^33^,^34^,^35^,^36^,^37^. The mechanism is similar to that of PDT and AVUT where microvessels are also destroyed by triggering the physiological functions of endothelial cells, red blood cells, and platelets. However, unlike PDT and AVUT which involve either photosensitizers or microbubbles, PUT is totally noninvasive and agent-free.

The most important advantage of the PUT technology is high precision, which features by its high selectivity on the treatment target. For targeted treatment on microvessels, the high selectivity of PUT is achieved by the high endogenous optical contrast between blood and other tissues in the visible to near-infrared spectrum. Additionally, the laser fluence needed by PUT is very low (as low as 4 mJ/cm^2^, which is under the safety limit set by American National Standards Institute (ANSI^38^) for skin exposure). In comparison, laser fluence used by photothermolysis is generally larger than 1 J/cm^2^. As a result, PUT may offer a certain penetration depth and precise localization for treating microvessels beyond the skin surface. The unique advantages of PUT, which is facilitated by synchronized light and sound irradiation, could be enormously significant when translating the technique to patients.

## Results

### Antivascular effect of PUT on single blood vessels

**Figure 1:**
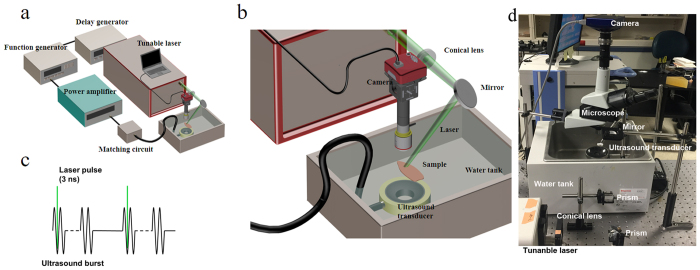
System schematic. (**a**,**b**) The PUT system consisted of a laser system and a therapeutic ultrasound system. A function generator was trigged by the laser system to generate burst signals at 1 MHz and 10% duty cycle with 10-Hz pulse repetition rate. The generated bursts were amplified by a 50-dB radio frequency amplifier before being sent to the therapeutic ultrasound transducer. Either an optical microscope or a PeriCam PSI System was employed for real-time monitoring of microcirculation. (**c**) Each laser pulse was delivered to the sample to overlay the beginning phase of each ultrasound burst. (**d**) Photograph of the system.

A PUT system is schematically illustrated in Fig. 1. A laser pulse (5-ns pulse width with 10-Hz pulse repetition rate) irradiated the target blood vessel with 532 nm light simultaneously with an ultrasound tone burst (1 MHz, 10% duty cycle, and 10-Hz pulse repetition rate). The applied ultrasound burst had a negative peak pressure of 0.45 MPa, and the laser fluence varied from 2 mJ/cm^2^ to 8 mJ/cm^2^. The treatment duration was either 4 minutes or until the target blood vessel was disrupted during the treatment, whichever occurred first. The posterior vitelline veins (p.v. veins) in chicken *yolk sac* membranes (embryo development day (EDD)-3) were selected as the target blood vessels because (1) they have a relatively uniform vessel size (150–200 μm), and (2) they barely have branches or adjacent vessels. For reference, Supplementary Figure 1 shows a photograph of a chicken yolk sac membrane with the main branches of veins and arteries labeled.

**Figure 2:**
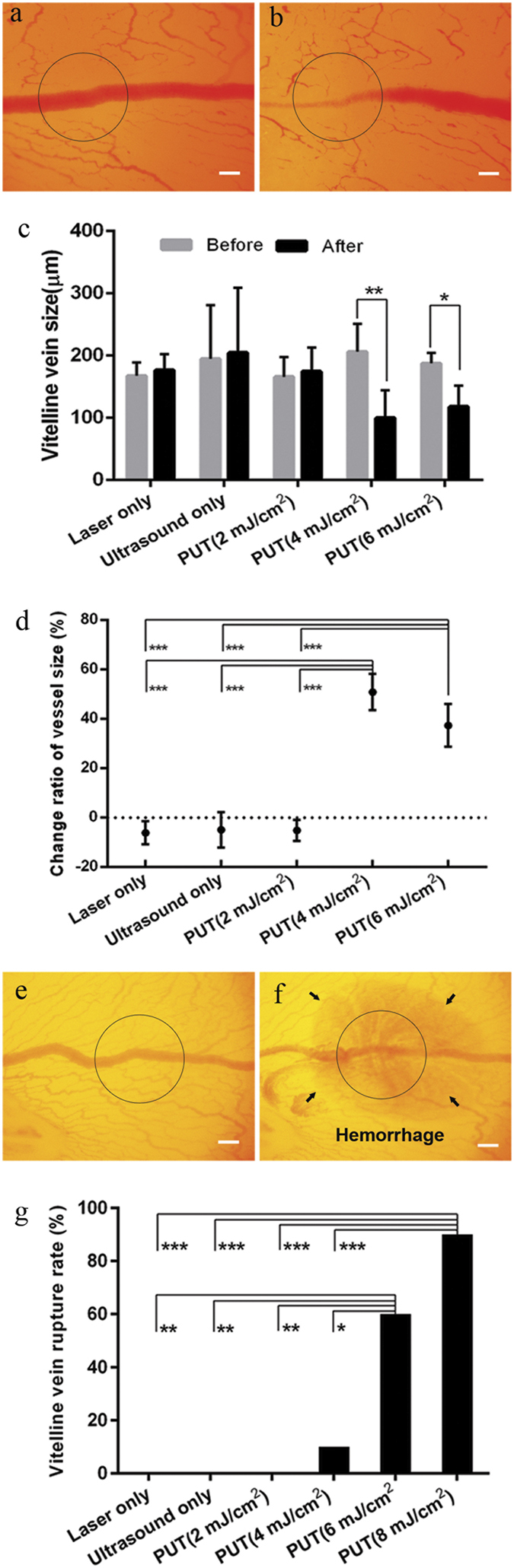
PUT on single blood vessels (veins) on chicken *yolk sac* membrane. (**a**,**b**) The photographs of a vessel before and after treatment, respectively. The vessel was treated by PUT with 0.45 MPa ultrasound negative peak pressure and 4 mJ/cm^2^ laser fluence at 532 nm. (**c**) Changes in blood vessel diameters after different treatments, including laser only (no ultrasound), ultrasound only (no laser), ultrasound and laser at 2 mJ/cm^2^ fluence, ultrasound and laser at 4 mJ/cm^2^ fluence, and ultrasound and laser at 6 mJ/cm^2^ fluence. Vertical bars demonstrate the standard deviation. (**d**) The relative change of the vitelline vein diameters [(D_before_ −D_after_)/D_before,_ where D_before_ is the diameter before treatment, and D_after_ is the diameter after treatment]. It shows that statistical significances exist between either of the two PUT groups (4 mJ/cm^2^ and 6 mJ/cm^2^ laser fluence) and any of the other three groups (ultrasound only, laser only, and PUT with 2 mJ/cm^2^ laser fluence). (**e**,**f)** The photographs of a vessel disrupted by PUT with 8 mJ/cm^2^ laser fluence at 532 nm. (**g**) Rate of vessel disruption for each treatment group. It shows that, in comparison with other groups, the disruption rates were remarkably high for PUT with 6 mJ/cm^2^ and 8 mJ/cm^2^ laser fluence. The applied laser fluence for laser-only groups: 20 mJ/cm^2^. The applied ultrasound negative peak pressure: 0.45 MPa, n = 10 for each group. The treated area is circled. *p < 0.05; **p < 0.01, ***p < 0.001. Scale bar: 200 μm.

Figure 2 shows the PUT treatment results on the p.v. veins of the chick embryo model *in vivo*. Figure 2a,b show the photographs of a p.v. vein before and after PUT. A significant reduction in the diameter of the vein was induced by the treatment. Figure 2c shows the quantified changes in the vein diameters before and after different treatments, including laser-only at 20 mJ/cm^2^ fluence (no ultrasound), ultrasound-only at 0.45 MPa negative peak pressure (no laser), ultrasound and laser at 2 mJ/cm^2^ fluence, ultrasound and laser at 4 mJ/cm^2^ fluence, and ultrasound and laser at 6 mJ/cm^2^ fluence. When either laser or ultrasound was applied alone, the changes in vessel diameters were insignificant. When laser and ultrasound were applied concurrently (i.e. PUT) with laser fluence of 2 mJ/cm^2^, no significant change in vessel diameter was noticed after the treatment. When the laser fluence was increased to 4 mJ/cm^2^ or 6 mJ/cm^2^, the reductions in the vessel diameters after PUT treatment became statistically significant (*p* < 0.01 for both 4 mJ/cm^2^ and 6 mJ/cm^2^, for paired t-test). Figure 2d shows the relative changes in vein diameters [(D_before_ − D_after_)/D_before,_ where D_before_ is the vessel diameter before the treatment, and D_after_ is the vessel diameter after the treatment]. The average reductions in the vessel diameter were 51% at 4 mJ/cm^2^ (p = 0.001, paired t test) and 37% at 6 mJ/cm^2^ (p = 0.018, paired t test). The difference between these two reductions, however, was statistically insignificant.

The disruption of the p.v. veins, or vessel rupture, during PUT was also investigated. Figure 2e,f show photographs of a disrupted vein induced by PUT. Figure 2g shows that the disruption of a blood vessel by PUT only became prominent when the laser fluence was increased to or above 6 mJ/cm^2^, with 60% of vessel disrupted at 6 mJ/cm^2^ and 90% at 8 mJ/cm^2^. At 4 mJ/cm^2^ laser fluence, the disruption rate was 10%. No blood vessel disruption occurred at either 2 mJ/cm^2^ laser fluence or in the control groups (i.e. ultrasound only and laser only). The differences between either of the two PUT treatment groups (6 mJ/cm^2^ and 8 mJ/cm^2^) and any of the rest of four groups (ultrasound only, laser only, PUT at 2 mJ/cm^2^, and PUT at 4 mJ/cm^2^) were statistically significant (p < 0.05, Fisher’s exact test).

### Selective antivascular effect of PUT on arteries and veins

PUT can selectively be used to treat arteries or veins based on the absorption spectrum of hemoglobin in the visible to near-infrared spectrum. By utilizing the difference in optical absorption spectra between oxy-hemoglobin (HbO_2_) and deoxy-hemoglobin (HbR), PUT can selectively treat either veins or arteries. We chose two optical wavelengths, 578 nm and 650 nm, to target HbO_2_ and HbR, respectively. The ratio between the optical absorption of HbO_2_ and HbR (HbO_2_/HbR) reaches a local maximum of 1.42 at 578 nm, while the ratio between the optical absorption of HbR and HbO_2_ (HbR/HbO_2_) reaches a local maximum of 10.2 at 650 nm. For chicken embryos, the oxygen saturation is opposite of normal adults, namely the vein has a high oxygen saturation and the artery a low oxygen saturation. The lateral vitelline vein (l.v. vein) is approximately 2.3 times the oxygen saturation of the lateral vitelline artery (l.v. artery)^39^. Therefore, PUT with 578-nm laser light would selectively treat l.v. veins, while PUT with 650-nm laser light would selectively treat l.v. arteries.

**Figure 3:**
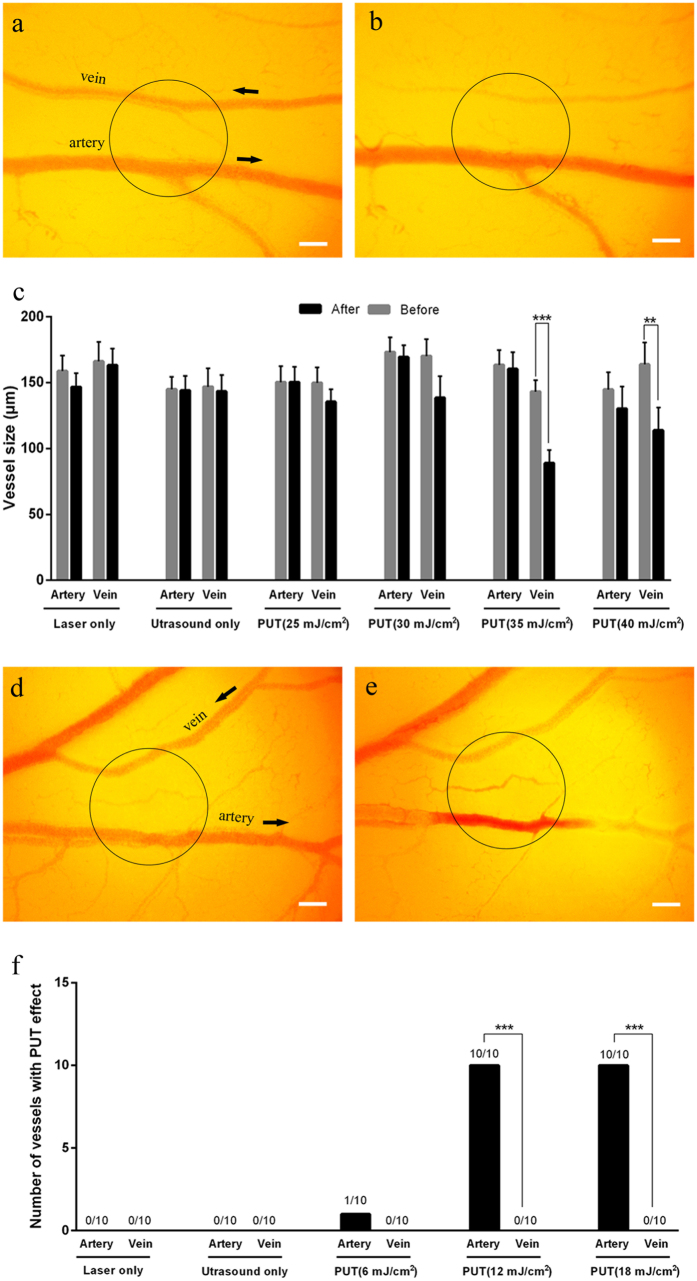
Selective treatment of veins or arteries on chicken *yolk sac* membranes using PUT. (**a**,**b**) The photographs of a pair of artery and vein before and after a treatment, respectively. PUT was performed with 0.25 MPa ultrasound negative peak pressure and 35 mJ/cm^2^ laser fluence at 578 nm. (**c**) Changes in the vessel diameters after different treatments. The changes of the vessel size were only statistically significant in the veins of the PUT groups with 35 mJ/cm^2^ and 40 mJ/cm^2^ laser fluence (n = 10 for each group). Vertical bars indicate standard deviation. The laser wavelengths in (**c**) were all at 578 nm. (**d**,**e**) The photographs of a vein/artery pair before and after a treatment, respectively. The PUT treatment was performed with 0.45 MPa ultrasound negative peak pressure and 12 mJ/cm^2^ laser fluence at 650 nm. (**f**) The number of veins and arteries that responded to each treatment (n = 10 for each group). Response to treatment was defined as either rupture or occlusion of a blood vessel. The laser wavelengths in (**f**) were all at 650 nm. The treated area is circled and black arrows indicate the orientation of blood flow. **p < 0.01; ***p < 0.001. Scale bar: 200 μm.

During PUT with 578-nm laser light targeting oxygenated Hb in the l.v. veins, blood vessel pairs consisting of a single vein and a single artery were selected as the treatment area. The applied ultrasound peak negative pressure was 0.25 MPa and the range of laser fluence was from 25 mJ/cm^2^ to 40 mJ/cm^2^. After 6 minutes of PUT with 35 mJ/cm^2^ and 40 mJ/cm^2^ laser fluence, significant vein thinning was observed (*p* < 0.001 for paired t-test) (Fig. 3a–c; and Supplementary Video 1). For the treatment groups of PUT at 20 mJ/cm^2^ and PUT at 30 mJ/cm^2^, mild vein thinning was noted, although the changes were statistically insignificant. For the two control groups, including laser-only with 40 mJ/cm^2^ fluence and ultrasound-only, no change in the vein diameters was observed. There was no change in artery diameter for any group using 578-nm laser (Fig. 3c). The relative reduction in the vein diameter in each group was also calculated, as shown in Supplementary Figure 2. Statistical significances were found when comparing the outcomes from the PUT groups with laser fluence ≥30 mJ/cm^2^ with those from the control groups (ultrasound only and laser only).

During PUT with 650-nm laser light targeting deoxygenated Hb in the l.v. arteries, similar blood vessel pairs were exposed to PUT with an ultrasound peak negative pressure of 0.45 MPa. At this wavelength, arteries were selectively treated. In our experiments, we noticed that the responses of arteries and veins were different after PUT. While veins typically decreased in size, arteries tended to develop blood clots or disrupt in addition to changing size. Therefore, the antivascular effect of PUT was defined as either rupture or occlusion of a blood vessel caused by PUT. After a 6-minute treatment, in the groups of PUT with 12 mJ/cm^2^ and 18 mJ/cm^2^ laser fluence, the antivascular effects were selectively induced to arteries while the vein in each blood vessel pair remained unaffected (n = 10, *p* < 0.001, Fisher’s exact test) (Fig. 3d–f, and Supplementary Video 2). The detailed statistical results are shown in Supplementary Figure 3. In the two control groups, where only laser (40 mJ/cm^2^) or only ultrasound (0.45 MPa peak negative pressure) was applied, no change in either veins or arteries was observed (*p* < 0.001 and *p* < 0.001 when compared with PUT with 12 mJ/cm^2^ and 18 mJ/cm^2^ groups, Fisher’s exact test), as shown in Fig. 3f. Moreover, in the group of PUT with 12 mJ/cm^2^ laser fluence, 80% of arteries (8 out of 10) were occluded and 20% (2 of 10) were disrupted. When the laser fluence was increased to 18 mJ/cm^2^, 50% of arteries were occluded and the other 50% were disrupted.

### Effect of PUT on rabbit auricular blood vessels *in vivo*

**Figure 4:**
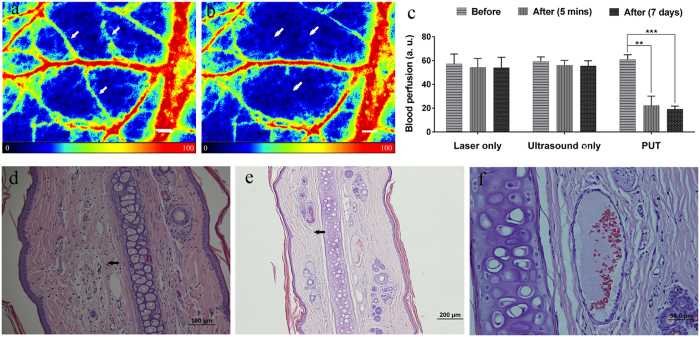
Effect of PUT on a rabbit ear model *in vivo* and histological photographs of rabbit ear tissues. (**a**,**b**) Examples of the blood perfusion maps measured by a PeriCam PSI System before and after PUT on a rabbit ear. The perfusion map clearly demonstrated diminished blood flow in the treated microvessels (white arrows) after the treatment. Scale bar: 1000 μm. (**c**) Averaged blood perfusion rate in the target microvessels before and after PUT treatment in 5 rabbits. **p < 0.01, ***p < 0.001. The applied ultrasound negative peak pressure was 0.45 MPa at 1 MHz with 10% duty cycle, and the laser fluence was 20 mJ/cm^2^ at 584 nm. (**d**) H&E stain image of rabbit ear tissue without PUT treatment, showing a normal auricular microvessel with a monolayer of integrated endothelial cells, basement membrane, and vascular smooth muscle (scale bar: 50 μm). (**e**,**f**) H&E stain images of rabbit ear tissue immediately following and 7 days after PUT treatment (scale bar: 50 μm), respectively. The formation of fibrin clot (black arrow) within the lumen in an auricular vein can be noticed, while the surrounding cells outside the blood vessel are not damaged.

Rabbit auricular blood vessels were treated with PUT with a peak negative ultrasound pressure of 0.45 MPa and a surface laser fluence of 20 mJ/cm^2^ at 584 nm. The blood perfusion rate in the treated region was monitored by a PeriCam PSI System, which is capable of real-time microcirculation imaging and evaluation of blood perfusion. Figure 4a,b show examples of the blood perfusion maps acquired before and after PUT on a rabbit ear *in vivo*. This example clearly demonstrates the diminishing of microcirculation in the treated region after PUT. To quantify the reduction in the blood perfusion, the perfusion rate in target microvessels was compared before and after treatment. Figure 4c shows the averaged reduction of perfusion rate from 5 rabbits. On average, a 63.5 ± 12% (p < 0.001, n = 5) reduction in perfusion rate was induced in the target microvessels. Histology confirmed that fibrin clots developed in the microvessels in the treated region, as shown in Fig. 4d,e, while no change occurred in the microvessels from the control groups (i.e. ultrasound-only and laser-only). Moreover, the histology results also demonstrate that, for all the samples treated with PUT, no damage was found in the surrounding tissue in rabbits euthanized either immediately following PUT treatment or 7-days after PUT treatment (Fig. 4f). This clearly indicates that the treatment effect of PUT was limited to the microvessels. In addition, the results show that PUT is effective for microvessels at a depth over 1 mm in scattering media *in vivo*.

**Figure 5:**
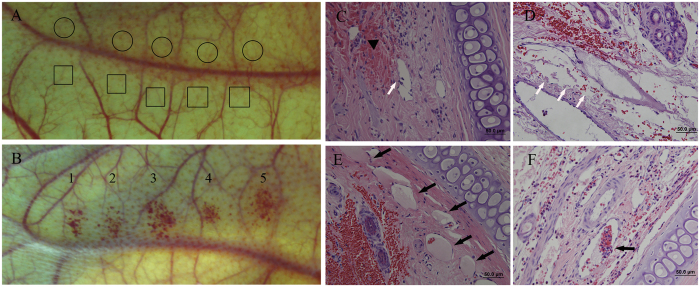
Photographs and histological results showing vessel disruption after PUT treatment. (**A**) The photograph of a control rabbit ear treated with either laser (circled area) or ultrasound (squared area) only. 1 MHz ultrasound-only at 0.6 MPa negative peak pressure (control) and with laser-only at 20 mJ/cm^2^ fluence at 584 nm (control). (**B**) The photograph of a rabbit ear after PUT treatment with 0.6 MPa ultrasound negative peak pressure and 20 mJ/cm^2^ laser fluence at 584 nm. Disruption of capillaries can be clearly noticed. (**C**–**F**) Histological photographs (H&E stain) showing the changes after the PUT treatment. (**C**) Shows detached endothelial cells from vessel wall (white arrow) and extravasation of erythrocytes (black triangle). (**D**) Shows disappearance of endothelial cells (white arrows). (**E**) Shows intravascular thrombus (black arrow). (**F**) Shows intravascular fibrin clot within the lumen and surrounding adherent red blood cells.

When the peak negative ultrasound pressure was increased from 0.45 MPa to 0.6 MPa while keeping the laser parameters the same, immediate disruption of microvessels was observed (100%) with an example result shown in Fig. 5A–D. Figure 5A shows the results from control groups (ultrasound-only and laser-only) while Fig. 5B is a photograph of a rabbit ear after PUT, demonstrating the local hemorrhage as a result of the treatment. The disruption of microvessels can be noticed in histology photographs after hematoxylin and eosin (H&E) stain, as shown in Fig. 5C,D. Clots in treated microvessels can also be found, as shown in Fig. 5E,F. In comparison, the results from the control groups did not show any sign of microvascular damage, as demonstrated by Fig. 5A and Supplementary Figure 4.

### Cavitation detection during PUT

**Figure 6:**
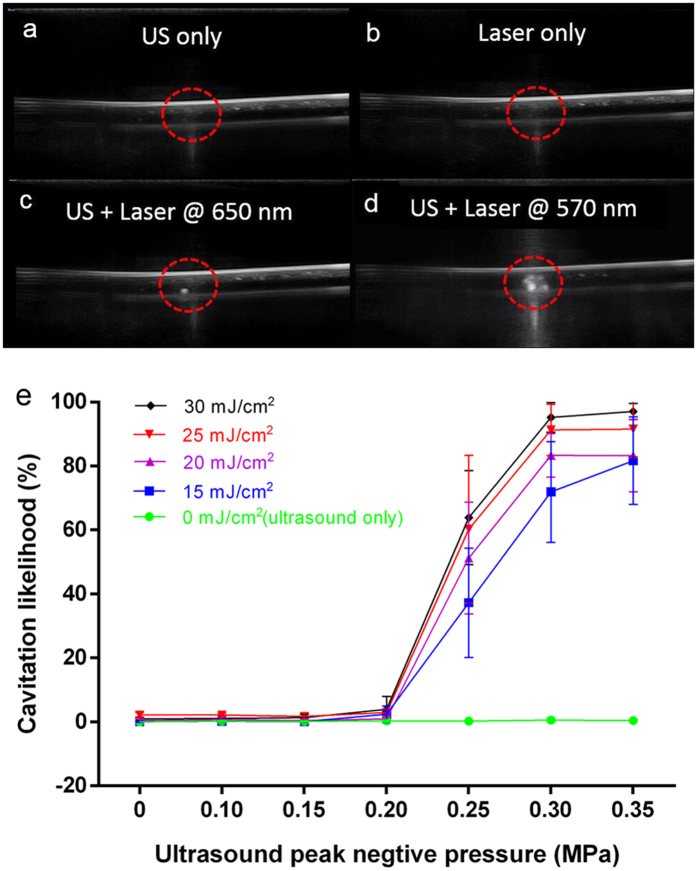
Studying the cavitation likelihood during PUT using a B-Mode ultrasound imaging system and a passive cavitation detector. Human whole blood in a 3-mm diameter IV tubing was treated. (**a**) Ultrasound image when therapeutic ultrasound was applied to the IV tubing alone (ultrasound-only). (**b**) Ultrasound image when laser was applied to the IV tubing alone (laser-only). (**c**,**d**) Ultrasound images when therapeutic ultrasound and laser were applied concurrently (PUT). Cavitation was clearly observed when PUT was applied, while no cavitation was observed when either ultrasound alone or laser alone was applied. It also shows that cavitation activity during PUT is highly dependent on the laser wavelength. The applied ultrasound negative peak pressure was 0.35 MPa at 1 MHz with 4% duty cycle, and the laser fluence was 20 mJ/cm^2^ for both wavelengths (650 nm and 570 nm). (**e**) Cavitation likelihood detected in the human whole blood by using a passive cavitation detector during the PUT treatment. The laser wavelength was 570 nm.

To better understand cavitation as the underlying mechanism of PUT, a study was performed using *ex vivo* human whole blood. The blood in a plastic intravenous (IV) tubing was treated with either ultrasound alone (control) or laser alone (control) or both laser and ultrasound (PUT). During the treatment, B-mode real-time ultrasound imaging was performed to visualize cavitation produced in the IV tubing. The results from the B-mode ultrasound imaging, as examples shown in Fig. 6, confirmed the enhanced cavitation activity produced by concurrently applied laser and ultrasound during PUT. Large number of bubbles were observed only when the laser pulses overlaid the ultrasound bursts, whereas neither the treatment involving only laser nor the treatment involving only ultrasound produced any detectable cavitation. We also found that the cavitation signal produced by the 570-nm laser was significantly stronger than that produced by the 650-nm laser at the same light fluence, because the optical absorption of the whole blood is more than 40 times higher at 570 nm than 650 nm. This demonstrates that PUT is a “spectroscopic” treatment technique. The PUT treatment outcome can be controlled by applying laser at different optical wavelengths. The original real-time B-mode ultrasound imaging clips can be found in Supplement Video 3 and 4.

We also quantified the likelihood of cavitation by employing a passive cavitation detector (PCD). The system schematic can be found in Supplementary Figure 5. Figure 6e shows the detected cavitation likelihood as functions of the ultrasound peak negative pressure and the laser fluence. When the laser fluence was 0 mJ/cm^2^ (ultrasound only), or ultrasound peak negative pressure was low (less than 0.2 MPa), the likelihood of cavitation was nearly 0%. For any laser fluence from 15 mJ/cm^2^ to 30 mJ/cm^2^, strong cavitation was detected only when the ultrasound peak negative pressure was larger than 0.2 MPa which seems to be a threshold. When the ultrasound peak negative pressure was larger than 0.3 MPa, the likelihood of cavitation approached a constant, suggesting that the cavitation activity became saturated in this range. Similar types of cavitation behavior have been observed previously and can be explained by the availability of cavitation nuclei in the blood^40^. By comparing PUT and the controls (laser only and ultrasound only), significant increases in cavitation likelihood were observed. This study on well-controlled *ex vivo* human blood specimens suggests that the cavitation, as the underlying mechanism of PUT, can be reliably produced by changing the laser and the ultrasound parameters.

## Discussion

We report for the first time our development of a novel technique termed PUT. PUT involves concurrently using safe light and safe ultrasound to achieve highly selective, precise treatment of microvessels without requiring an exogenous agent. The high precision of PUT is closely related to its underlying mechanism which is photoacoustic cavitation, or more generally, the photospallation effect^41^,^42^. When pulsed laser energy is absorbed by blood, photospallation may produce cavitation through strong, transient thermal-elastic stress^41^,^43^. Moreover, in an object with spherical or cylindrical shape (e.g. a blood vessel), the laser-induced photoacoustic wave can converge into the center and achieve a significantly high acoustic pressure and produce cavitation^44^, which is referred to as “cold bubbles” and has been observed in cells^45^. Unlike other use of therapeutic ultrasound and laser irradiation^46^,^47^,^48^,^49^,^50^, when ultrasound and laser irradiation are concurrently applied, a portion of the ultrasound waves can modulate the transient thermal-elastic negative stress produced through photospallation, which will increase the cavitation efficiency because of the greater negative pressure^31^. One of the key features of photoacoustic cavitation is that cavitation may be induced at a laser energy level that is much lower than that needed for laser thermal therapies (such as photothermolysis and photocoagulation) because photoacoustic cavitation is not a strictly thermal effect. Moreover, the synergistic application of ultrasound will further reduce the laser energy needed for cavitation.

PUT is highly selective to a treatment target (e.g. a blood vessel) and demonstrates excellent controllability on the desired outcome. In PUT, the cavitation in the treated tissue is initiated by the optical energy deposition of the laser pulses in the tissue. Strong cavitation occurs only within an area of high optical absorption where the ultrasound beam and laser beam overlap. The high selectivity of PUT is enabled by the endogenous optical absorption variation between various tissue types. Different tissues contain different concentrations of various chromophores, and have largely different optical absorption spectra which are their unique “optical fingerprints”. The optical fingerprint can facilitate the differentiation of tissue types with optical techniques that are highly sensitive and specific. As an example, working at the wavelength(s) enabling the best optical absorption contrast of HbO_2_ or HbR over other chromophores, PUT shows the unique capability to selectively self-target either arteries or veins. Since blood absorbs much more optical energy than the surrounding tissue, cavitation is limited to blood vessels and, hence, the treatment is highly localized. Since the powers of both applied ultrasound and laser pulses in PUT are low, neither ultrasound-alone nor laser-alone can produce tissue damage, leaving the surrounding tissue intact. The excellent controllability of PUT is demonstrated by its capability to control the magnitude, spatial extent, and duration of cavitation produced in the target tissue (i.e. a blood vessel) by adjusting the optical and ultrasonic parameters. PUT can activate different treatment mechanisms in a target vessel, either through disruption or embolization, through precise control of the cavitation dynamics. These two mechanisms both have clinical value, and both can be realized by PUT operating with different laser and ultrasound parameters.

Anti-VEGF antibodies have gained great popularity to treat angiogenesis during the last decade. However, anti-VEGF antibodies solely neutralize the cytokine VEGF, and anti-VEGF therapy is limited^51^,^52^,^53^. This is indicated by a number of studies that initial antitumor effects relapse or anti-angiogenesis yield part of patients with no or poor respond after the use of VEGF inhibitors^2^,^15^,^16^,^17^,^54^,^55^. Furthermore, frequent administration of anti-VEGF drugs also was associated with direct annual medical cost^56^,^57^. Thus, developing alternative or adjunct therapies to the current anti-VEGF drugs has drawn increasing attention. In the current study, the findings on rabbit ear model *in vivo* suggest that PUT can treat microvessels beyond the surface of optically scattering tissues (depth of microvessels: ~500 μm). Because laser energy significantly scatters in tissue, the therapeutic effects of photothermolysis and photocogulation are limited to very superficial surface layer^58^. Although the PUT not yet fully studied, we expect that its treatment depth would be comparable to PDT, and would be deeper than conventional laser ablation, since neither PDT nor PUT relies on photo-thermal effect as in laser ablation. Additionally, Unlike PDT, PUT do not require the injection of exogenous agents. Therefore, most of the conditions that can be treated by PDT and laser ablation, including superficial tumors such as head and neck cancer^59^, melanoma^60^, and capillary malformation such as port-wine stain^61^, and retinal diseases such as polypoidal choroidal vasculopathy, choroidal neovascularization, and central serous chorioretinopathy^62^, could potentially benefit from the development and application of PUT. In addition, current clinical applications which involve focal laser therapy of the macula, such as diabetic macular edema and branch retinal vein occlusions, would be other potential clinical applications.

To better implement PUT in a clinical setting, particularly when the target vessel is deep in soft tissue, a high-resolution vascular imaging will be needed to guide the treatment. Photoacoustic imaging (PAI) technique can be naturally combined with PUT, as PUT and PAI can share the same nanosecond pulse duration laser. PAI could benefit PUT treatment planning by detecting the morphological and functional information in the target sample, and by measuring the local light fluence and optical absorption information of vessels and background tissue. PAI could also help to evaluate the treatment efficacy by measuring the changes in vessel size and oxygen saturation in the treated vessels^63^. All of this information about the subsurface tissue can be acquired at excellent spatial and temporal resolution by PAI, and would be essential to facilitate personalized treatment and optimized treatment outcomes.

In summary, a hybrid ultrasound and optical technique, PUT, has been developed for antivascular therapy. The acute antivascular effect of the technique has been verified through the studies on a chicken embryo model and a rabbit ear model. PUT holds significant potential as a novel non-invasive, agent-free, highly selective, and highly controllable antivascular treatment.

## Methods

### PUT system

The schematic of the PUT system which integrates a laser system with a therapeutic ultrasound system is demonstrated in Fig. 1. A standard Nd:YAG pumped optical parameter oscillator (OPO) system (Surelite OPO PLUS, Continuum, San Jose, CA) was employed to produce laser pulses with 5-ns pulse duration and 10-Hz pulse repetition rate. The laser beam was delivered to the sample with its energy on the sample surface measured by a Nova PE25BB-SH-V2 pyroelectric head (Ophir Optronics Ltd., Jerusalem, Israel). The laser spot size was measured using knife-edge techniques (~8 mm in diameter) and laser intensity was carefully adjusted by changing the Q-switch. A 1-MHz therapeutic ultrasound transducer (H102, Sonic Concepts, Bothell, WA) was used to simultaneously supply ultrasound bursts with the pulsed laser. The therapeutic transducer has a geometric focal distance of 62.6 mm, a focal depth of 13.5 mm, and a focal width of 1.33 mm. The transducer was mounted to a 3-dimentional translation stage so that its focal spot can be adjusted to target the sample and overlay the laser spot. The peak negative ultrasound pressure was measured *in situ* by using a calibrated needle hydrophone (HNC-1500, Onda, Sunnyvale, CA). Both the ultrasound transducer and samples were immersed in a water bath which was heated to 37.5 °C during each experiment. Either an optical microscope (M300, AmScope, Irvine, CA) or a PeriCam PSI System (Perimed, Järfälla, Sweden) was employed for real-time monitoring of the treatment effect.

The laser system supplied laser pulses at various power levels. Each laser pulse was delivered to the sample to overlay the beginning phase of each ultrasound burst (Fig. 1). A function generator (HP33250A, Agilent Technologies, Santa Clara, CA) was trigged by the laser system to produce 1 MHz bursts (10-ms long) of 10% duty cycle at a pulse repetition rate of 10 Hz. The signals from the function generator were then amplified by a 50-dB radio frequency amplifier (240 L, ENI Technology, Inc., Rochester, NY) before being sent to the therapeutic ultrasound transducer. Cavitation occurs when the laser pulse and the beginning of the ultrasound burst are synergistically overlaid. The remaining portion of the ultrasound burst further drives the formed bubbles to stimulate the endothelium in the blood vessels.

### *In vivo* chicken embryo model

Fertilized chicken eggs were obtained from a local farm (Townline Poultry Farm, Inc., Zeeland, MI). Culturing of chick embryo followed the protocol in the literatures^64^,^65^. In brief, eggs were cleaned with 70% ethanol and then hatched in a 38.0 °C humidified incubator. On embryo development day (EDD) 3, eggs were removed from the incubator, cleaned again with 70% ethanol, gently cracked, and then the chick embryos were transferred to a sterilized petri dish. The bottom of the petri dish was cut to form a 6 cm-diameter hole which was covered by a piece of plastic wrap (Glad Cling Wrap) to allow ultrasound bursts to propagate through. In each group, 10 chicken embryos were included. For the experiment of single vessel treatment, the posterior vitelline veins (p.v. vein) were selected as the target vessels. In the experiment showing the selective treatment of veins or arteries, pairs of vein and artery on the *yolk sac* membrane were selected and covered by the treatment zone.

During the treatment, the flow of red blood cells in microvessels could be clearly videotaped through a 4× objective lens by using the optical microscope with 25 frames/s. Before and after each treatment, angiographic images of the chicken embryos were also captured by the optical microscopy using the 4× objective lens. Using the National Institutes of Health (NIH) Image J software program, the vessels widths were quantified by counting the pixels covered by the vessels.

### *In vivo* rabbit ear model

New Zealand white rabbits (2.5 to 3.0 kg, 6 to 8 weeks old, both genders) were acquired from the Center for Advanced Models for Translational Sciences and Therapeutics at the University of Michigan Medical School. All the animal handling procedures were carried out according to NIH guidelines and an animal protocol approved 9/3/2015 by the Institutional Animal Care and Use Committee at the University of Michigan, protocol number PRO00006487, PI Paulus. Briefly, each rabbit was anesthetized with 3% to 4% isoflurane. Respiratory rate, heart rate, response to stimuli, and temperature were monitored. After achieving anesthesia, both rabbit ears were shaved to facilitate better ultrasound coupling. Then, the rabbit was placed on a warm platform with one ear placed in the water tank horizontally. The ear was mounted to a custom-built holder allowing flexible and reliable positioning of the treatment region. A PeriCam PSI System (Perimed, Järfälla, Sweden) was employed for real-time monitoring during treatment. Before and after each treatment, auricular microcirculation maps were also captured by the PeriCam PSI system.

At the end of each experiment, the rabbit was euthanized with a lethal dose of isoflurane along with induction of bilateral pneumothorax and removal of a vital organ to ensure the animals would not revive. The auricular vessel segments were then dissected and placed in fixative (10% buffered formalin) solution for 3 days and then embedded in paraffin. The paraffin-embedded sections (thickness = 5 μm) cut vertically to the treated vessels were stained with hematoxylin and eosin (H&E). Slides were deparaffinized and underwent a series of xylene and alcohol wash. Histology photographs were taken with an Olympus BX-51 microscope, and then analyzed by an experienced pathologist (Dr. John E. Wilkinson), who was blind to the treatment procedures.

### Cavitation detection in *ex vivo* blood specimens

Human whole blood was obtained from the University of Michigan Blood Center. Before each treatment, a plastic intravenous (IV) tubing (inner diameter 3 mm; outer diameter 4 mm) which is soft and optically transparent was immersed in a water bath filled with 37.5 °C water, and placed through the focal zone of the therapeutic transducer. Blood was injected into the plastic IV tubing by using a syringe. The laser beam overlaid the focal zone of the ultrasound transducer. A Zonare ZS3 ultrasound imaging system (Zonare Medical Systems, Inc., Mountain View, CA) running on the B-mode was used to monitor the cavitation produced in the tubing in real time.

To quantitatively evaluate cavitation events, a passive cavitation detector (PCD)^66^,^67^ was used to detect the signals generated from the focal zone of the therapeutic ultrasound transducer. The PCD was orientated at a 90 degree angle from the therapeutic ultrasound transducer, and its focal zone overlaid the focal zone of the therapeutic ultrasound (Supplementary Figure 5). We used a broadband 20-MHz center frequency focused ultrasound transducer (V316, Olympus-NDT, Waltham, MA) so that the detection was more sensitive to the high frequency broadband noise. In addition, a high pass filter with a cutoff frequency of 5 MHz was used to minimize the interference signal from the 1 MHz therapeutic ultrasound. The detected signal was amplified 60 dB before being collected by a personal computer through a GPIB controlled digital oscilloscope (TDS540, Tektronix, Inc., Beaverton, OR). A cavitation event was identified when the amplitude of the detected signal was at least twice of that of the noise baseline.

### Statistical analysis

Statistical analysis was performed using SPSS software version 19.0 (SPSS Inc., Chicago, IL). Paired t test was used to compare vessel size of chicken embryos and blood perfusion before and after treatment in the same group. The Student’s t test was applied for comparisons of independent data, and the Fisher’s exact test or *χ*^2^ test for comparisons of categorical data between groups. Data are expressed as mean ± standard error of the mean. A 2-tailed P-value of <0.05 was used to indicate the statistical significance.

## Additional Information

**How to cite this article:** Hu, Z. *et al*. High-precision, non-invasive anti-microvascular approach via concurrent ultrasound and laser irradiation. *Sci. Rep.* **7**, 40243; doi: 10.1038/srep40243 (2017).

**Publisher's note:** Springer Nature remains neutral with regard to jurisdictional claims in published maps and institutional affiliations.

## Supplementary Material

Supplementary Information

Supplementary video 1

Supplementary video 2

Supplementary video 3

Supplementary video 4
