# Supplementary material for: High-precision, non-invasive anti-microvascular approach via concurrent ultrasound and laser irradiation
Source: Sci Rep. 2017 Jan 11;7:40243. doi: 10.1038/srep40243 (PMC5225605; doi:10.1038/srep40243)
Supplement: Supplementary Information [file srep40243-s1.pdf]

Supplementary Information

High-precision, non-invasive anti-microvascular approach via concurrent ultrasound and laser irradiation

Zizhong Hu[1,2,3], Haonan Zhang[4], Aghapi Mordovanakis[2], Yannis M. Paulus[1,2], Qinghuai Liu[3], Xueding Wang[1,4], and Xinmai Yang[5]

[1]Department of Biomedical Engineering, University of Michigan, Ann Arbor, MI, USA

[2]Department of Ophthalmology and Visual Sciences, University of Michigan, Ann Arbor, MI, USA

[3]Department of Ophthalmology, the First Affiliated Hospital of Nanjing Medical University, Nanjing, P.R. China.

[4]Department of Radiology, University of Michigan, Ann Arbor, MI, USA

[5]Bioengineering Research Center and Department of Mechanical Engineering, University of Kansas, Lawrence, KS, USA

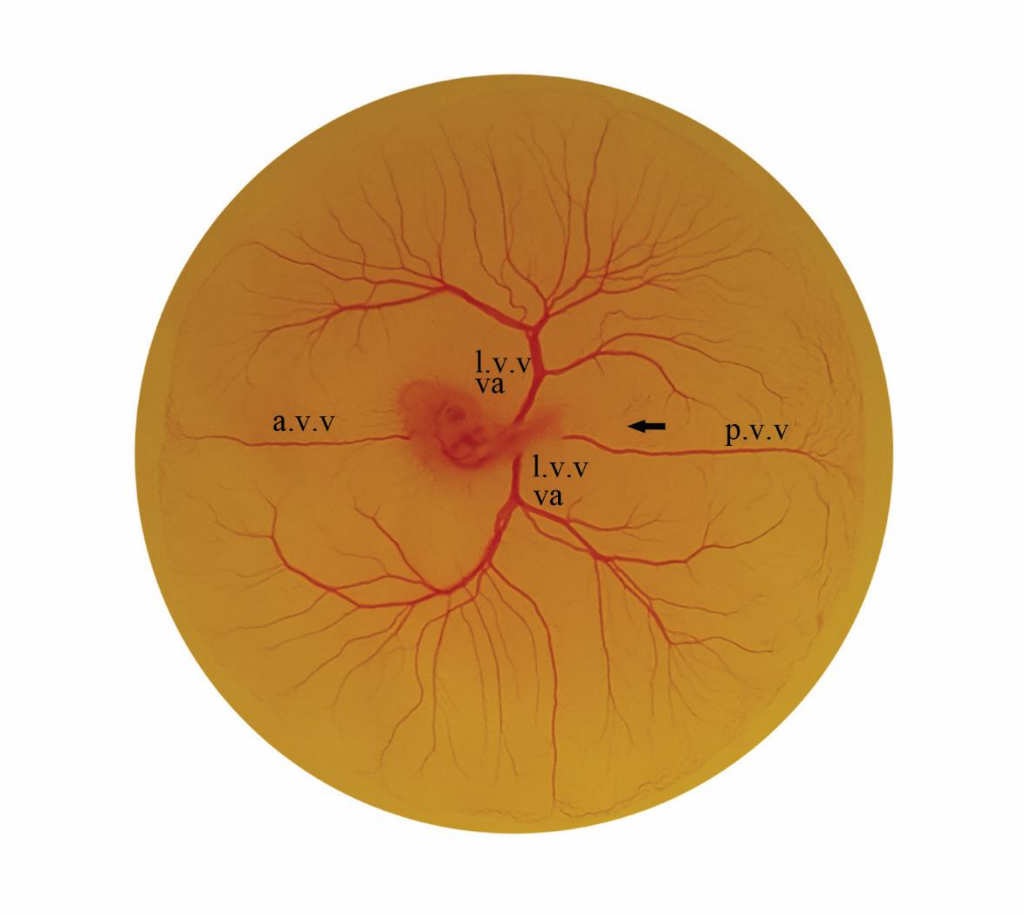

Supplementary Figure 1 | Photograph of an embryo development day 3 (EED 3) chicken *yolk sac* membrane with the main branches of veins and arteries labelled.

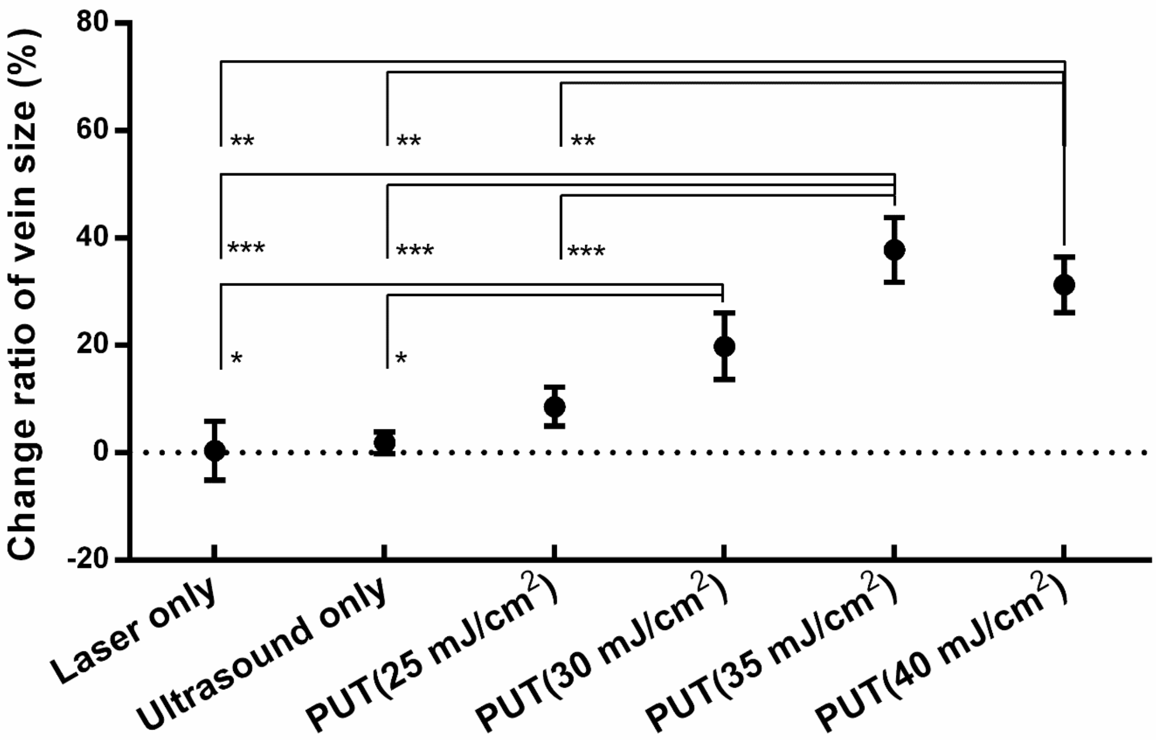

Supplementary Figure 2 | The relative reductions in the vein diameter after different treatments. The reduction ratio is defined as $(D_{before} - D_{after})/D_{before}$, where D_{before} is the diameter before treatment, and D_{after} is the diameter after treatment. For the laser-only group, the applied laser fluence was 40 mJ/cm^2. For the ultrasound-only group, the applied ultrasound negative peak pressure was 0.45 MPa. PUT was performed with different laser fluences (25 mJ/cm^2, 30 mJ/cm^2, 35 mJ/cm^2, and 40 mJ/cm^2) at 578-nm wavelength. $^{*}p < 0.05$; $^{**}p < 0.01$; $^{***}p < 0.001$.

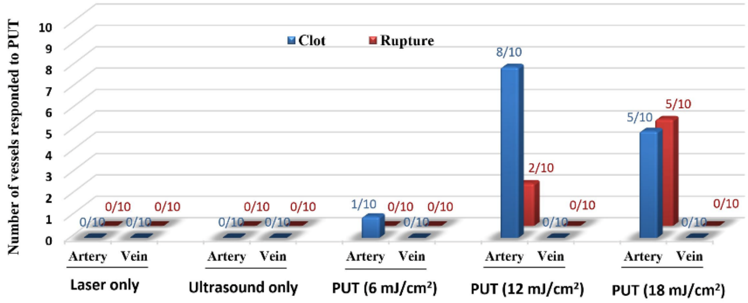

Supplementary Figure 3 | Antivascular effects induced by different treatments. For the laser-only group, the applied laser fluence was 40 mJ/cm^2. For the ultrasound-only group, the applied ultrasound negative peak pressure was 0.45 MPa. PUT was performed with different laser fluences (6 mJ/cm^2, 12 mJ/cm^2, and 18 mJ/cm^2) at 650-nm wavelength.

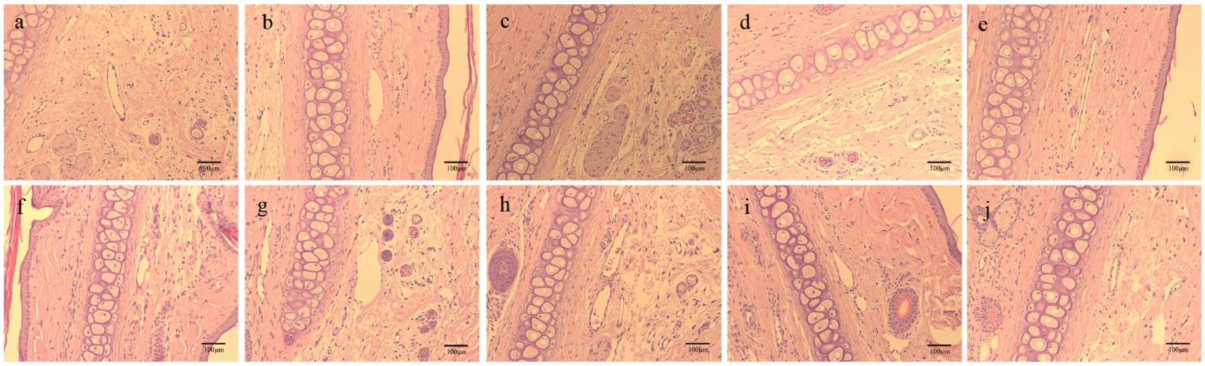

Supplementary Figure 4 | Histological results from the two control groups in the rabbit ear experiment. (a-e) The results from the ultrasound-only group (0.45 MPa negative peak pressure at 1 MHz with 10% duty cycle). (f-j) The results from the laser only group (20 mJ/cm^2 at 584 nm). No vascular damage was noticed in either of the two control groups. Scale bar: 100 µm.

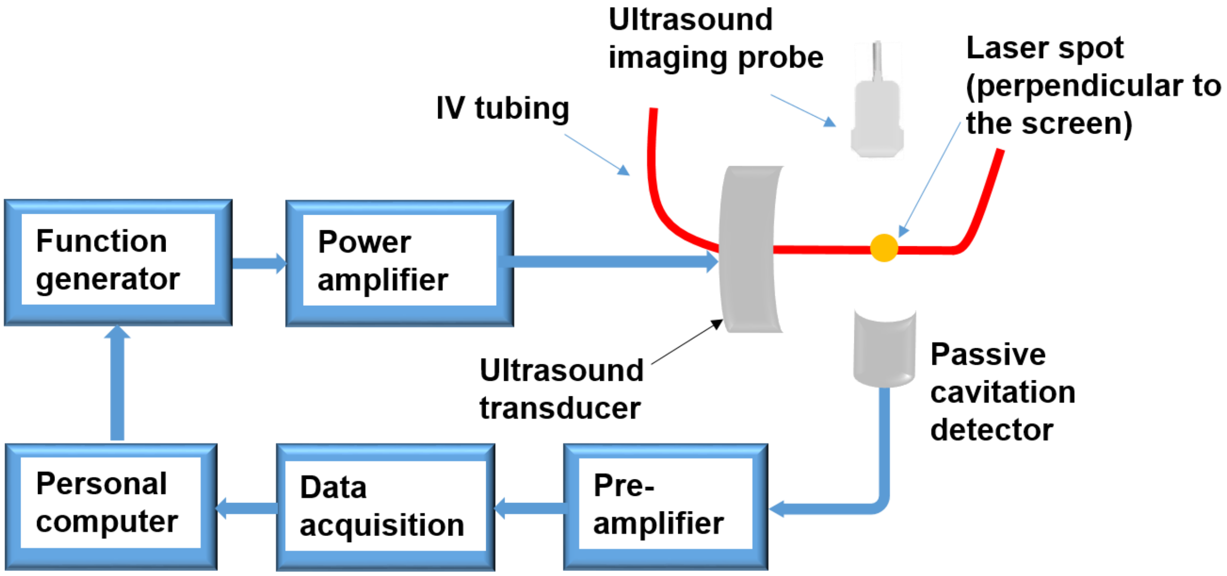

Supplementary Figure 5 | Schematic of the system for cavitation detection. This system was used to evaluate the cavitation activity during the PUT treatment on *ex vivo* human blood.

Supplementary Video 1 | Effect of PUT treatment on a vein/artery pair with 578-nm laser light. Vein shrinkage was induced while no change occurred in artery. Arrows indicate the directions of blood flows. The applied ultrasound negative peak pressure was 0.25 MPa and the laser fluence was 35 mJ/cm^2 at 578 nm.

Supplementary Video 2 | Effect of PUT treatment on a vein/artery pair with 650-nm laser light. A clot was induced in the artery while no change occurred in the vein. Arrows indicate the directions of blood flows. The applied ultrasound negative peak pressure was 0.45 MPa and the laser fluence was 12 mJ/cm^2 at 650 nm.

Supplementary Video 3 | Cavitation activity induced by PUT with 570-nm laser in *ex vivo* human whole blood. Video clips were captured by a Zonare ZS3 B-mode ultrasound imaging system. Cavitation activity was only produced when laser and therapeutic ultrasound were applied synergistically. The applied therapeutic ultrasound negative peak pressure was 0.35 MPa and the laser fluence was 20 mJ/cm^2 laser at 570 nm.

Supplementary Video 4| Cavitation activity induced by PUT with 650-nm laser in *ex vivo* human whole blood. Cavitation clips was captured by a Zonare ZS3 B-mode ultrasound imaging system. Cavitation activity was only produced when laser and therapeutic ultrasound were applied synergistically. The applied ultrasound pressure and the laser fluence were the same as those in Supplementary video 3. Due to the lower optical absorption of the blood specimen at 650 nm, the cavitation activity was much weaker than that induced by PUT with 570 nm laser.
